# Host‐induced silencing of a nematode chitin synthase gene enhances resistance of soybeans to both pathogenic *Heterodera glycines* and *Fusarium oxysporum*

**DOI:** 10.1111/pbi.13808

**Published:** 2022-03-29

**Authors:** Lingan Kong, Xue Shi, Deng Chen, Nan Yang, Changfa Yin, Jun Yang, Gaofeng Wang, Wenkun Huang, Huan Peng, Deliang Peng, Shiming Liu

**Affiliations:** ^1^ State Key Laboratory for Biology of Plant Diseases and Insect Pests Institute of Plant Protection Chinese Academy of Agricultural Sciences Beijing China; ^2^ 34752 College of Plant Protection China Agricultural University Beijing China; ^3^ Institute of Plant Protection Jiangxi Academy of Agricultural Sciences Nanchang China

**Keywords:** Soybean, nematode chitin synthase, host‐induced gene silencing, *Heterodera glycines*, *Fusarium oxysporum*, resistance

Soybean cyst nematode (SCN), *Heterodera glycines*, is a devastating pathogen in soybean worldwide, causing huge yield losses annually. Host‐induced gene silencing (HIGS) has shown potentials to control plant parasitic nematodes by generating transgenic plants carrying hairpin constructs against nematode target genes (Chaudhary et al., 2019; Li *et al*., 2010; Mani *et al*., 2020; Shivakumara *et al*., 2017; Tian *et al*., 2016). Chitin synthesized by chitin synthase (Chs) is present in fungi and nematodes but absent in plants and vertebrate animals and is a target in *Magnaporthe oryzae* and *Fusarium graminearum* for designing new fungicides and developing novel resistant varieties by HIGS (Cheng *et al*., 2015; Kong *et al*., 2012). Here, we mainly aimed to develop heritable SCN *CHS*‐HIGS soybeans conferring enhanced SCN resistance. Meanwhile, the developed HIGS soybeans were tested for resistance to fungus *Fusarium oxysporum*, which causes soybean Fusarium wilt disease.

First, the sole *CHS* gene (*SCN‐CHS*) with 3984 bp (GenBank Acc. No.: OK149168) was cloned from SCN HG Type 1.2.3.5.7 (race 4, SCN4). SCN‐Chs contained a typical chitin synthase catalytic domain (Chs) and seven transmembrane domains (TMs) (Figure 1a). qRT‐PCR analysis indicated that *SCN‐CHS* was highly expressed at egg stage when compared to other developmental stages (Figure 1b). A 420 bp cDNA fragment of *SCN‐CHS* catalytic region positioned at 1936–2355 bp was used to generate transgenic HIGS soybeans employing ‘Jack’ as wild‐type soybean (Figure 1c), and three homozygous transgenic lines (‘48‐7‐5’, ‘55‐8‐24’, and ‘57‐9‐2’) with yellow seed‐coat identical to that of Jack (Figure 1d) were obtained. The amount of SCN4 cysts per plant (left of Figure 1e) and eggs per cyst (Figure 1f) in T2 lines was all significantly reduced when compared to Jack. The cyst numbers among T2 lines differed dramatically from 18 to 76, while the cyst numbers were about 120 in Jack, on the average, and the T2 line ‘55‐8‐24(T2)’ showed the most inhibited SCN cyst formation with a 6‐fold reduction in cyst numbers (left of Figure 1e). These results indicated that various HIGS lines suppressed SCN cyst formation differently. The average numbers of eggs per cyst were 153 in T2 lines, which were significantly decreased by 37.5% when compared to that in Jack (Figure 1f). qRT‐PCR analysis showed that *SCN‐CHS* expression in eggs of SCN parasitizing in T2 lines was significantly reduced (Figure 1g). These results suggested that down‐regulation of *SCN‐CHS* expression in eggs was likely associated with suppression of SCN cyst and egg formation in T2 soybeans. Analyses of the time‐course developmental progress of SCN4 juveniles in T2 roots showed that all juveniles were decreased, and the developmental transition frequency from low to high molt stages was also delayed in all T2 roots (Figure 1h). What is more, the size of cysts formed in T2 lines ranged from 662.33 to 673.92 μm in diameter and was obviously smaller than that in Jack (703.4–724.24 μm) (Figure 1i).

**Figure 1:**
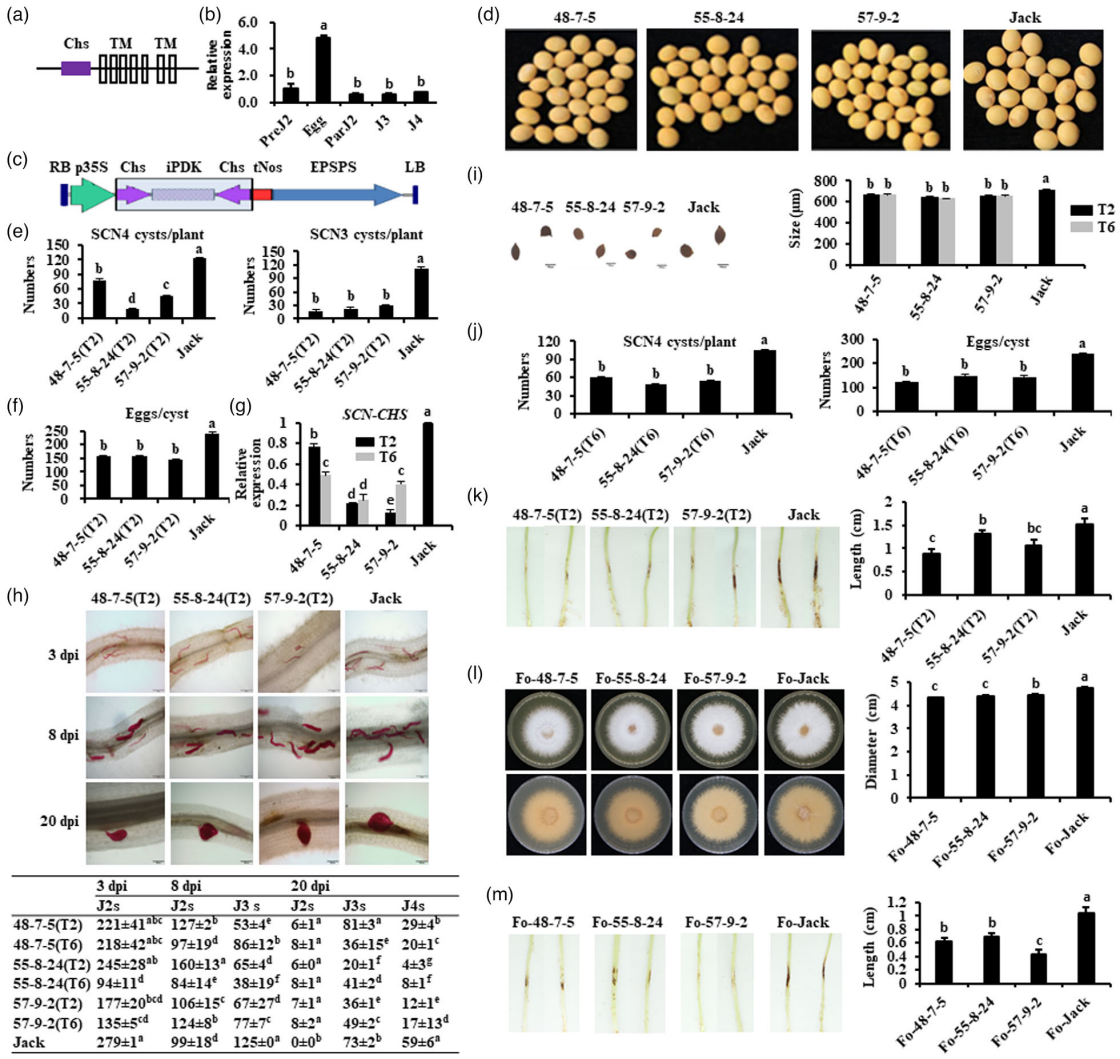
Development of enhanced broad‐spectrum and durable resistance of soybeans against nematode *Heterodera glycines* and fungus *Fusarium oxysporum* by host‐induced silencing of the soybean cyst nematode chitin synthase gene *SCN‐CHS*. (a) Domain structure of SCN *Heterodera glycines* chitin synthase (SCN‐Chs). (b) Expression of *SCN‐CHS* in SCN. PreJ2, preparasitic second‐stage juvenile; ParJ2, parasitic J2. (c) Map of HIGS construct. (d) Seeds of T2 HIGS soybeans (48‐7‐5, 55‐8‐24, and 57‐9‐2) and wild‐type Jack. (e) Phenotype of T2 HIGS soybeans and Jack infected with SCN HG Type 1.2.3.5.7 (race 4, SCN4) or SCN HG Type 0 (race 3, SCN3). (f) Numbers of eggs per cyst in roots infected with SCN race 4. (g) Expression of *SCN‐CHS* in eggs of SCN race 4 infecting T2/T6 HIGS soybeans. T2 and T6 denote T2 and T6 generations, respectively. *HgActin* was used as the reference gene. (h) Development of juveniles in roots infected by hatched SCN race 4 J2s. (i) Shape (left) and size (right) of cysts in T2 HIGS lines and Jack. (j) Numbers of cysts per HIGS soybean plant (left) and eggs per cyst (right) in T6 HIGS soybeans and Jack. (k) Hypocotyl phenotype of T2 HIGS soybeans infected by *Fusarium oxysporum* f. sp. *glycinces*. (l) Mycelia growth of *F*. *oxysporum* isolated from T2 HIGS soybeans and Jack. Fo‐48‐7‐5, Fo‐55‐8‐24, Fo‐57‐9‐2, and Fo‐Jack represent *F*. *oxysporum* isolated from T2 HIGS soybeans, and Jack, respectively. (m) Phenotype of Jack hypocotyls inoculated with *F*. *oxysporum* isolated from T2 HIGS soybeans and Jack. All the relevant data were statistically analysed with five replicates using SPSS v25 software. Different letters denote significant difference at *P*<0.05 using Duncan’s new multiple range test.

Meanwhile, a similar but stronger effectiveness on the suppression of cyst formation was observed in all the SCN HG Type 0 (race 3, SCN3)‐infected T2 lines (right of Figure 1e). These results together with SCN4 infection results implied that T2 HIGS soybean lines expressing dsRNA of *SCN‐CHS* showed enhanced broad‐spectrum resistance to different SCN HG types (races).

The heredity of HIGS soybeans suppressing SCN was evaluated using three T6 lines, ‘48‐7‐5(T6)’, ‘55‐8‐24(T6)’, and ‘57‐9‐2 (T6)’, with infection of SCN4. The amount of both cysts per plant and eggs per cyst was also significantly decreased in all T6 lines (Figure 1j). Moreover, expression of *SCN‐CHS* in SCN eggs was also obviously suppressed in all T6 lines (Figure 1g). All these results suggested that T6 HIGS lines exhibited strong heredity of the boosted resistance to SCN.

Subsequently, the three HIGS soybean lines were tested for their resistance against *Fusarium oxysporum* f. sp. *glycines*. The longer the lesions are on the hypocotyls, the more serious the disease is. The results showed that the average hypocotyl lesion length in all HIGS lines was significantly decreased when compared to that in Jack (Figure 1k). Afterwards, *F. oxysporum* was isolated from each HIGS line and Jack as Fo‐48‐7‐5, Fo‐55‐8‐24, Fo‐57‐9‐2, and Fo‐Jack, respectively, and they were then cultured on PDA‐medium plates and mycelia growth was observed. About 1 week post‐inoculation, the average spread size (diameter) of mycelia isolated from all the HIGS soybeans was remarkably reduced when compared to that isolated from Jack (Figure 1l). Meanwhile, after these isolated fungi inoculating Jack, the lesions of hypocotyls infected by Fo‐48‐7‐5, Fo‐55‐8‐24, or Fo‐57‐9‐2 were all significantly shorter than these infected by Fo‐Jack (Figure 1m). These results indicated that HIGS of *SCN‐CHS* significantly enhanced soybean resistance against *F. oxysporum*.

In summary, our study showed that host‐induced silencing of chitin synthase gene in SCN (*SCN‐CHS*) broadly and durably enhanced soybean resistance against both different SCN races and *F*. *oxysporum*, and the developed *SCN‐CHS* HIGS soybeans will be a potential for controlling SCN and Fusarium wilt diseases.

## Conflict of interest

The authors declare no conflict of interest.

## Author contributions

LK, SL, JY, and DP designed the experiments. LK, XS, DC, NY, CY, JY, SL, GW, WH, and HP performed the experiments and data analyses. LK and SL wrote the manuscript. All the authors participated in discussion and revision.
